# Di-*tert*-butyl 2,6,11-trioxo-2,3-dihydro-1*H*-anthra[1,2-*d*]imidazole-1,3-diacetate

**DOI:** 10.1107/S1600536811022914

**Published:** 2011-06-18

**Authors:** Zahra Afrakssou, Yousef Kandri Rodi, Natalie Saffon, El Mokhtar Essassi, Seik Weng Ng

**Affiliations:** a Laboratoire de Chimie Organique Appliquée, Faculté des Sciences et Techniques, Université Sidi Mohamed Ben Abdallah, Fés, Morocco; b Service Commun Rayons-X FR2599, Université Paul Sabatier Bâtiment 2R1, 118 route de Narbonne, Toulouse, France; c Laboratoire de Chimie Organique Hétérocyclique, Pôle de Compétences Pharmacochimie, Université Mohammed V-Agdal, BP 1014 Avenue Ibn Batout, Rabat, Morocco; d Department of Chemistry, University of Malaya, 50603 Kuala Lumpur, Malaysia

## Abstract

The fused-ring system of the title compound, C_27_H_28_N_2_O_7_, which comprises one five- and three six-membered rings, is approximately planar (r.m.s. deviation = 0.133 Å), the system being buckled along the axis passing through the O atoms of the anthraquinone portion of the mol­ecule. Within the anthraquinone portion, the two benzene rings are aligned at 7.3 (2)°. In the crystal, one of the *tert*-butyl groups is disordered over two sets of sites in a 1:1 ratio. Weak inter­molecular C—H⋯O hydrogen bonding is present in the crystal structure.

## Related literature

For a related structure, see: Afrakssou *et al.* (2010 ▶).
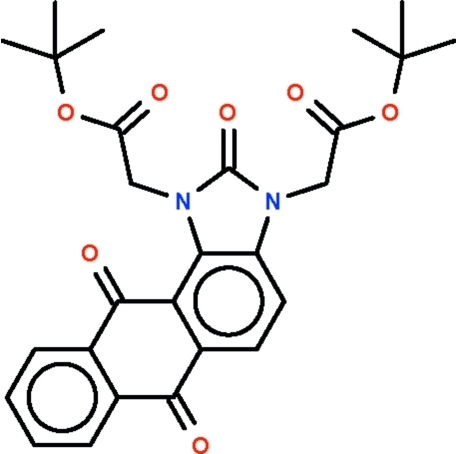


## Experimental

### 

#### Crystal data

C_27_H_28_N_2_O_7_
*M* *_r_* = 492.51
Monoclinic, 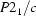

*a* = 19.5785 (4) Å
*b* = 13.0330 (3) Å
*c* = 9.9269 (2) Å
β = 90.583 (1)°
*V* = 2532.88 (9) Å^3^
*Z* = 4
Mo *K*α radiation
μ = 0.09 mm^−1^
*T* = 293 K
0.50 × 0.10 × 0.10 mm

#### Data collection

Bruker APEXII diffractometer
37371 measured reflections
5172 independent reflections
2929 reflections with *I* > 2σ(*I*)
*R*_int_ = 0.070

#### Refinement

*R*[*F* ^2^ > 2σ(*F* ^2^)] = 0.056
*wR*(*F* ^2^) = 0.190
*S* = 1.04
5172 reflections
353 parameters
48 restraints
H-atom parameters constrained
Δρ_max_ = 0.51 e Å^−3^
Δρ_min_ = −0.31 e Å^−3^

### 

Data collection: *APEX2* (Bruker, 2005 ▶); cell refinement: *SAINT* (Bruker, 2005 ▶); data reduction: *SAINT*; program(s) used to solve structure: *SHELXS97* (Sheldrick, 2008 ▶); program(s) used to refine structure: *SHELXL97* (Sheldrick, 2008 ▶); molecular graphics: *X-SEED* (Barbour, 2001 ▶); software used to prepare material for publication: *publCIF* (Westrip, 2010 ▶).

## Supplementary Material

Crystal structure: contains datablock(s) global, I. DOI: 10.1107/S1600536811022914/xu5244sup1.cif

Structure factors: contains datablock(s) I. DOI: 10.1107/S1600536811022914/xu5244Isup2.hkl

Supplementary material file. DOI: 10.1107/S1600536811022914/xu5244Isup3.cml

Additional supplementary materials:  crystallographic information; 3D view; checkCIF report

## Figures and Tables

**Table 1:** Hydrogen-bond geometry (Å, °)

*D*—H⋯*A*	*D*—H	H⋯*A*	*D*⋯*A*	*D*—H⋯*A*
C16—H16*B*⋯O2^i^	0.97	2.54	3.320 (4)	137
C22—H22*A*⋯O5^i^	0.97	2.47	3.391 (3)	159
C22—H22*B*⋯O7^i^	0.97	2.29	3.190 (4)	154

Symmetry code: (i) 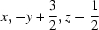.

**Fig. 1.**
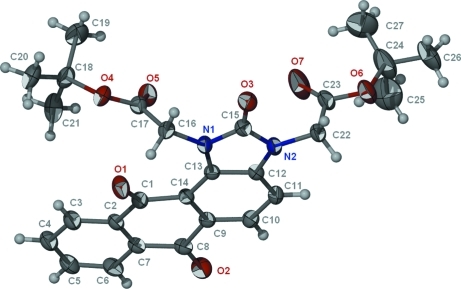
Thermal ellipsoid plot (Barbour, 2001) of C27H28N2O7 at the 50% probability level; hydrogen atoms are drawn as arbitrary radius. The disorder is not shown.

## supplementary crystallographic information

### Comment

A recent study reported 1,3-diallyl-1*H*-anthra[1,2-*d*]imidazole-2,6,11(3*H*)-trione (Afrakssou *et al.*, 2010). The title compound has in place of the allyl group the *tert*-butyl acetate ester group. The fused-ring system of C_27_H_28_N_2_O_7_ (Scheme I) that comprises one five-membered ring and three six-membered rings is appromimately planar, the system being buckled along the axis passing through the O atoms of the anthraquinone portion of the molecule. With the anthraquinone portion, the two benzene rings are aligned at 7.3 (2) ° (Fig. 1).

### Experimental

To a solution of 1*H*-anthra [2,1-*d*] imidazole-2, 6,11(3*H*)-trione (0.3 g, 1.13 mmol), potassium carbonate (0.62 g, 4.53 mmol) and tetra *n*-butyl ammonium bromide (0.03 g, 0.018 mmol) in DMF (15 ml) was added *tert*-butyl bromoacetate (0.56 ml, 0.47 mmol). Stirring was continued at room temperature for 24 h. The mixture was filtered and the solvent removed. The residue was extracted with water. The organic compound was chromatographed on a column of silica gel with ethyl acetate/hexane. Orange prismatic crystals were isolated when the solvent was allowed to evaporate.

### Refinement

Carbon-bound H-atoms were placed in calculated positions (C—H 0.93–0.97 Å) and were included in the refinement in the riding model approximation, with *U*(H) set to 1.2–1.5*U*(C).

One of the two *t*-butyl butyl groups is disordered in the methyl units; the disorder could not be refined, and was assumed to be a 1:1 type of disorder. The C–C_methyl_ distances were restrained to 1.54±0.01 Å and the C_methyl_–C_methyl_ distances to 2.51±0.01 Å; the anisotropic temperature factors were restrained to be nearly isotropic.

Omitted from the refinment because of bad disagreements were (1 0 0) and (-3 1 2).

### Crystal data

**Table:**

C_27_H_28_N_2_O_7_	*F*(000) = 1040
*M**_r_* = 492.51	*D*_x_ = 1.292 Mg m^−^^3^
Monoclinic, *P*2_1_/*c*	Mo *K*α radiation, λ = 0.71073 Å
Hall symbol: -P 2ybc	Cell parameters from 5392 reflections
*a* = 19.5785 (4) Å	θ = 2.6–23.5°
*b* = 13.0330 (3) Å	µ = 0.09 mm^−^^1^
*c* = 9.9269 (2) Å	*T* = 293 K
β = 90.583 (1)°	Prism, orange
*V* = 2532.88 (9) Å^3^	0.50 × 0.10 × 0.10 mm
*Z* = 4	

### Data collection

**Table:**

Bruker APEXII diffractometer	2929 reflections with *I* > 2σ(*I*)
Radiation source: fine-focus sealed tube	*R*_int_ = 0.070
graphite	θ_max_ = 26.4°, θ_min_ = 1.9°
φ and ω scans	*h* = −24→24
37371 measured reflections	*k* = −16→16
5172 independent reflections	*l* = −12→12

### Refinement

**Table:**

Refinement on *F*^2^	Secondary atom site location: difference Fourier map
Least-squares matrix: full	Hydrogen site location: inferred from neighbouring sites
*R*[*F*^2^ > 2σ(*F*^2^)] = 0.056	H-atom parameters constrained
*wR*(*F*^2^) = 0.190	*w* = 1/[σ^2^(*F*_o_^2^) + (0.086*P*)^2^ + 1.5472*P*] where *P* = (*F*_o_^2^ + 2*F*_c_^2^)/3
*S* = 1.04	(Δ/σ)_max_ = 0.001
5172 reflections	Δρ_max_ = 0.51 e Å^−^^3^
353 parameters	Δρ_min_ = −0.31 e Å^−^^3^
48 restraints	Extinction correction: *SHELXL97* (Sheldrick, 2008), Fc^*^=kFc[1+0.001xFc^2^λ^3^/sin(2θ)]^-1/4^
Primary atom site location: structure-invariant direct methods	Extinction coefficient: 0.0068 (12)

### Fractional atomic coordinates and isotropic or equivalent isotropic displacement parameters (Å^2^)

**Table:**

	*x*	*y*	*z*	*U*_iso_*/*U*_eq_	Occ. (<1)
O1	0.11503 (11)	0.55465 (16)	0.3982 (2)	0.0529 (6)	
O2	0.09491 (12)	0.94796 (16)	0.5443 (2)	0.0529 (6)	
O3	0.33296 (11)	0.57044 (16)	0.0875 (2)	0.0468 (6)	
O4	0.21612 (11)	0.36402 (15)	0.3971 (2)	0.0435 (5)	
O5	0.26519 (12)	0.50970 (17)	0.4779 (2)	0.0504 (6)	
O6	0.46459 (11)	0.8677 (2)	0.1416 (2)	0.0636 (7)	
O7	0.41731 (15)	0.7703 (3)	0.3008 (3)	0.1046 (13)	
N1	0.24308 (12)	0.60746 (17)	0.2331 (2)	0.0347 (6)	
N2	0.30396 (12)	0.73659 (18)	0.1509 (2)	0.0365 (6)	
C1	0.11879 (14)	0.6422 (2)	0.4430 (3)	0.0368 (7)	
C2	0.07265 (14)	0.6734 (2)	0.5542 (3)	0.0358 (7)	
C3	0.03615 (15)	0.5987 (3)	0.6220 (3)	0.0452 (8)	
H3	0.0414	0.5302	0.5985	0.054*	
C4	−0.00819 (16)	0.6249 (3)	0.7246 (3)	0.0484 (8)	
H4	−0.0315	0.5743	0.7714	0.058*	
C5	−0.01722 (16)	0.7274 (3)	0.7564 (3)	0.0484 (8)	
H5	−0.0471	0.7455	0.8247	0.058*	
C6	0.01780 (15)	0.8034 (3)	0.6876 (3)	0.0450 (8)	
H6	0.0106	0.8721	0.7084	0.054*	
C7	0.06374 (14)	0.7765 (2)	0.5873 (3)	0.0357 (7)	
C8	0.10414 (14)	0.8571 (2)	0.5187 (3)	0.0381 (7)	
C9	0.15721 (14)	0.8242 (2)	0.4211 (3)	0.0336 (6)	
C10	0.19751 (15)	0.9009 (2)	0.3665 (3)	0.0392 (7)	
H10	0.1901	0.9687	0.3917	0.047*	
C11	0.24838 (15)	0.8789 (2)	0.2757 (3)	0.0402 (7)	
H11	0.2757	0.9304	0.2403	0.048*	
C12	0.25714 (14)	0.7778 (2)	0.2397 (3)	0.0335 (6)	
C13	0.21739 (13)	0.6969 (2)	0.2917 (3)	0.0307 (6)	
C14	0.16564 (13)	0.7197 (2)	0.3849 (3)	0.0320 (6)	
C15	0.29788 (15)	0.6315 (2)	0.1490 (3)	0.0377 (7)	
C16	0.22597 (15)	0.4999 (2)	0.2478 (3)	0.0357 (7)	
H16A	0.2529	0.4600	0.1852	0.043*	
H16B	0.1782	0.4901	0.2246	0.043*	
C17	0.23880 (15)	0.4609 (2)	0.3887 (3)	0.0384 (7)	
C18	0.21185 (18)	0.3115 (3)	0.5299 (3)	0.0516 (9)	
C19	0.2819 (2)	0.2970 (4)	0.5889 (5)	0.0877 (15)	
H19A	0.3091	0.2572	0.5283	0.132*	
H19B	0.2786	0.2618	0.6736	0.132*	
H19C	0.3029	0.3628	0.6028	0.132*	
C20	0.1781 (2)	0.2106 (3)	0.4936 (4)	0.0653 (11)	
H20A	0.2081	0.1713	0.4377	0.098*	
H20B	0.1361	0.2237	0.4458	0.098*	
H20C	0.1687	0.1729	0.5744	0.098*	
C21	0.1649 (2)	0.3734 (3)	0.6196 (4)	0.0678 (11)	
H21A	0.1866	0.4371	0.6431	0.102*	
H21B	0.1555	0.3352	0.7000	0.102*	
H21C	0.1228	0.3871	0.5723	0.102*	
C22	0.36054 (14)	0.7901 (2)	0.0903 (3)	0.0392 (7)	
H22A	0.3449	0.8556	0.0554	0.047*	
H22B	0.3778	0.7502	0.0157	0.047*	
C23	0.41684 (16)	0.8075 (3)	0.1919 (3)	0.0500 (8)	
C24	0.5229 (2)	0.9059 (4)	0.2242 (5)	0.0988 (18)	
C25	0.4904 (6)	0.9979 (8)	0.3039 (11)	0.111 (4)	0.50
H25A	0.4702	1.0455	0.2415	0.166*	0.50
H25B	0.5253	1.0319	0.3558	0.166*	0.50
H25C	0.4559	0.9722	0.3631	0.166*	0.50
C26	0.5707 (6)	0.9463 (9)	0.1166 (11)	0.088 (4)	0.50
H26A	0.5858	0.8905	0.0615	0.132*	0.50
H26B	0.6094	0.9785	0.1589	0.132*	0.50
H26C	0.5469	0.9956	0.0616	0.132*	0.50
C27	0.5567 (6)	0.8344 (8)	0.3191 (11)	0.113 (4)	0.50
H27A	0.5805	0.7825	0.2695	0.170*	0.50
H27B	0.5229	0.8026	0.3748	0.170*	0.50
H27C	0.5886	0.8715	0.3747	0.170*	0.50
C25'	0.5058 (7)	0.9391 (11)	0.3628 (9)	0.138 (5)	0.50
H25D	0.4839	0.8838	0.4093	0.207*	0.50
H25E	0.4757	0.9971	0.3586	0.207*	0.50
H25F	0.5469	0.9579	0.4102	0.207*	0.50
C26'	0.5574 (8)	0.9876 (8)	0.1401 (13)	0.102 (5)	0.50
H26D	0.5656	0.9613	0.0514	0.153*	0.50
H26E	0.6001	1.0064	0.1817	0.153*	0.50
H26F	0.5284	1.0469	0.1339	0.153*	0.50
C27'	0.5644 (6)	0.8040 (7)	0.2276 (13)	0.125 (4)	0.50
H27D	0.5753	0.7840	0.1373	0.187*	0.50
H27E	0.5376	0.7513	0.2692	0.187*	0.50
H27F	0.6058	0.8141	0.2785	0.187*	0.50

### Atomic displacement parameters (Å^2^)

**Table:**

	*U*^11^	*U*^22^	*U*^33^	*U*^12^	*U*^13^	*U*^23^
O1	0.0527 (14)	0.0363 (13)	0.0702 (16)	−0.0088 (10)	0.0218 (12)	−0.0110 (11)
O2	0.0533 (14)	0.0369 (13)	0.0689 (16)	0.0024 (10)	0.0138 (12)	−0.0128 (11)
O3	0.0468 (13)	0.0471 (13)	0.0468 (13)	0.0058 (10)	0.0136 (10)	−0.0021 (10)
O4	0.0575 (13)	0.0329 (11)	0.0400 (12)	−0.0022 (10)	0.0018 (10)	0.0040 (9)
O5	0.0556 (14)	0.0515 (14)	0.0441 (13)	−0.0146 (11)	−0.0043 (10)	−0.0006 (10)
O6	0.0410 (13)	0.095 (2)	0.0546 (15)	−0.0213 (13)	−0.0013 (11)	0.0134 (13)
O7	0.0606 (18)	0.197 (4)	0.0563 (18)	−0.043 (2)	−0.0125 (14)	0.055 (2)
N1	0.0357 (13)	0.0299 (12)	0.0385 (13)	−0.0027 (10)	0.0071 (10)	−0.0007 (10)
N2	0.0335 (13)	0.0361 (14)	0.0402 (14)	−0.0039 (10)	0.0071 (11)	0.0026 (10)
C1	0.0338 (15)	0.0302 (16)	0.0465 (17)	−0.0005 (12)	0.0034 (13)	−0.0010 (12)
C2	0.0269 (14)	0.0401 (16)	0.0404 (16)	−0.0015 (12)	0.0031 (12)	0.0001 (12)
C3	0.0384 (17)	0.0448 (18)	0.0526 (19)	−0.0030 (14)	0.0082 (14)	−0.0007 (14)
C4	0.0379 (17)	0.059 (2)	0.0490 (19)	−0.0024 (15)	0.0099 (15)	0.0031 (15)
C5	0.0353 (17)	0.062 (2)	0.0476 (19)	0.0006 (15)	0.0093 (14)	−0.0059 (16)
C6	0.0371 (17)	0.0472 (19)	0.0510 (19)	0.0030 (14)	0.0056 (14)	−0.0123 (15)
C7	0.0283 (15)	0.0378 (16)	0.0408 (16)	0.0007 (12)	0.0013 (12)	−0.0045 (12)
C8	0.0332 (15)	0.0371 (17)	0.0439 (17)	0.0015 (13)	−0.0020 (13)	−0.0062 (13)
C9	0.0297 (14)	0.0293 (15)	0.0417 (16)	−0.0003 (11)	−0.0014 (12)	−0.0023 (12)
C10	0.0399 (16)	0.0287 (15)	0.0489 (18)	−0.0008 (12)	0.0012 (14)	−0.0009 (12)
C11	0.0394 (16)	0.0329 (16)	0.0483 (18)	−0.0050 (13)	0.0043 (13)	0.0045 (13)
C12	0.0295 (14)	0.0347 (15)	0.0363 (15)	0.0004 (12)	0.0014 (12)	0.0012 (12)
C13	0.0287 (14)	0.0285 (14)	0.0348 (14)	−0.0002 (11)	−0.0012 (11)	−0.0012 (11)
C14	0.0282 (14)	0.0313 (15)	0.0366 (15)	−0.0011 (11)	0.0014 (11)	−0.0010 (11)
C15	0.0349 (15)	0.0402 (17)	0.0381 (16)	0.0004 (13)	0.0035 (13)	0.0017 (13)
C16	0.0419 (16)	0.0272 (14)	0.0382 (16)	0.0013 (12)	0.0048 (12)	−0.0029 (11)
C17	0.0384 (16)	0.0365 (17)	0.0405 (17)	−0.0022 (13)	0.0025 (13)	0.0001 (13)
C18	0.061 (2)	0.050 (2)	0.0438 (19)	−0.0059 (16)	−0.0049 (16)	0.0148 (15)
C19	0.071 (3)	0.097 (3)	0.095 (3)	−0.001 (2)	−0.019 (2)	0.048 (3)
C20	0.091 (3)	0.042 (2)	0.063 (2)	−0.0064 (19)	0.004 (2)	0.0163 (17)
C21	0.090 (3)	0.063 (2)	0.051 (2)	−0.023 (2)	0.018 (2)	−0.0003 (18)
C22	0.0337 (15)	0.0465 (18)	0.0377 (16)	−0.0033 (13)	0.0070 (13)	0.0050 (13)
C23	0.0335 (17)	0.073 (2)	0.0437 (19)	−0.0046 (16)	0.0079 (14)	0.0119 (16)
C24	0.052 (3)	0.162 (5)	0.082 (3)	−0.047 (3)	−0.014 (2)	0.022 (3)
C25	0.109 (7)	0.121 (8)	0.101 (7)	−0.027 (6)	−0.020 (6)	−0.020 (6)
C26	0.046 (5)	0.121 (8)	0.097 (7)	−0.038 (6)	0.004 (5)	−0.009 (6)
C27	0.082 (6)	0.139 (8)	0.118 (7)	−0.014 (6)	−0.057 (6)	0.018 (6)
C25'	0.135 (8)	0.171 (9)	0.109 (8)	−0.053 (8)	−0.027 (7)	−0.012 (7)
C26'	0.080 (7)	0.125 (9)	0.102 (8)	−0.050 (7)	−0.002 (6)	0.002 (7)
C27'	0.075 (6)	0.186 (9)	0.112 (8)	−0.012 (7)	−0.016 (6)	0.040 (7)

### Geometric parameters (Å, °)

**Table:**

O1—C1	1.227 (3)	C18—C19	1.498 (5)
O2—C8	1.225 (3)	C18—C20	1.512 (5)
O3—C15	1.220 (3)	C18—C21	1.519 (5)
O4—C17	1.341 (3)	C19—H19A	0.9600
O4—C18	1.488 (4)	C19—H19B	0.9600
O5—C17	1.203 (3)	C19—H19C	0.9600
O6—C23	1.322 (4)	C20—H20A	0.9600
O6—C24	1.484 (5)	C20—H20B	0.9600
O7—C23	1.186 (4)	C20—H20C	0.9600
N1—C13	1.399 (3)	C21—H21A	0.9600
N1—C15	1.402 (4)	C21—H21B	0.9600
N1—C16	1.449 (3)	C21—H21C	0.9600
N2—C15	1.374 (4)	C22—C23	1.503 (4)
N2—C12	1.387 (3)	C22—H22A	0.9700
N2—C22	1.445 (4)	C22—H22B	0.9700
C1—C14	1.485 (4)	C24—C27	1.477 (7)
C1—C2	1.490 (4)	C24—C25'	1.484 (7)
C2—C3	1.386 (4)	C24—C26'	1.516 (7)
C2—C7	1.394 (4)	C24—C26	1.521 (7)
C3—C4	1.387 (4)	C24—C27'	1.557 (8)
C3—H3	0.9300	C24—C25	1.574 (7)
C4—C5	1.384 (5)	C25—H25A	0.9600
C4—H4	0.9300	C25—H25B	0.9600
C5—C6	1.389 (4)	C25—H25C	0.9600
C5—H5	0.9300	C26—H26A	0.9600
C6—C7	1.393 (4)	C26—H26B	0.9600
C6—H6	0.9300	C26—H26C	0.9600
C7—C8	1.484 (4)	C27—H27A	0.9600
C8—C9	1.490 (4)	C27—H27B	0.9600
C9—C10	1.387 (4)	C27—H27C	0.9600
C9—C14	1.419 (4)	C25'—H25D	0.9600
C10—C11	1.380 (4)	C25'—H25E	0.9600
C10—H10	0.9300	C25'—H25F	0.9600
C11—C12	1.376 (4)	C26'—H26D	0.9600
C11—H11	0.9300	C26'—H26E	0.9600
C12—C13	1.412 (4)	C26'—H26F	0.9600
C13—C14	1.410 (4)	C27'—H27D	0.9600
C16—C17	1.507 (4)	C27'—H27E	0.9600
C16—H16A	0.9700	C27'—H27F	0.9600
C16—H16B	0.9700		
			
C17—O4—C18	120.7 (2)	H20A—C20—H20B	109.5
C23—O6—C24	122.3 (3)	C18—C20—H20C	109.5
C13—N1—C15	110.0 (2)	H20A—C20—H20C	109.5
C13—N1—C16	132.8 (2)	H20B—C20—H20C	109.5
C15—N1—C16	117.1 (2)	C18—C21—H21A	109.5
C15—N2—C12	109.7 (2)	C18—C21—H21B	109.5
C15—N2—C22	122.8 (2)	H21A—C21—H21B	109.5
C12—N2—C22	126.3 (2)	C18—C21—H21C	109.5
O1—C1—C14	121.8 (3)	H21A—C21—H21C	109.5
O1—C1—C2	119.2 (3)	H21B—C21—H21C	109.5
C14—C1—C2	119.0 (2)	N2—C22—C23	110.7 (2)
C3—C2—C7	119.7 (3)	N2—C22—H22A	109.5
C3—C2—C1	119.2 (3)	C23—C22—H22A	109.5
C7—C2—C1	121.0 (3)	N2—C22—H22B	109.5
C2—C3—C4	120.9 (3)	C23—C22—H22B	109.5
C2—C3—H3	119.6	H22A—C22—H22B	108.1
C4—C3—H3	119.6	O7—C23—O6	126.0 (3)
C5—C4—C3	119.2 (3)	O7—C23—C22	123.3 (3)
C5—C4—H4	120.4	O6—C23—C22	110.7 (3)
C3—C4—H4	120.4	C27—C24—O6	118.5 (5)
C4—C5—C6	120.7 (3)	C27—C24—C25'	72.3 (6)
C4—C5—H5	119.6	O6—C24—C25'	115.5 (6)
C6—C5—H5	119.6	C27—C24—C26'	126.5 (9)
C5—C6—C7	119.8 (3)	O6—C24—C26'	106.0 (7)
C5—C6—H6	120.1	C25'—C24—C26'	114.3 (7)
C7—C6—H6	120.1	C27—C24—C26	113.1 (6)
C2—C7—C6	119.6 (3)	O6—C24—C26	101.7 (6)
C2—C7—C8	120.3 (2)	C25'—C24—C26	134.1 (8)
C6—C7—C8	120.0 (3)	C26'—C24—C26	24.4 (7)
O2—C8—C7	120.6 (3)	C27—C24—C27'	38.5 (5)
O2—C8—C9	121.2 (3)	O6—C24—C27'	97.1 (6)
C7—C8—C9	118.3 (2)	C25'—C24—C27'	110.5 (6)
C10—C9—C14	121.6 (3)	C26'—C24—C27'	112.1 (6)
C10—C9—C8	116.8 (2)	C26—C24—C27'	89.2 (7)
C14—C9—C8	121.6 (2)	C27—C24—C25	109.9 (6)
C11—C10—C9	121.5 (3)	O6—C24—C25	102.7 (5)
C11—C10—H10	119.3	C25'—C24—C25	38.3 (6)
C9—C10—H10	119.3	C26'—C24—C25	85.8 (7)
C12—C11—C10	117.5 (3)	C26—C24—C25	110.1 (6)
C12—C11—H11	121.3	C27'—C24—C25	148.4 (6)
C10—C11—H11	121.3	C24—C25—H25A	109.5
C11—C12—N2	128.4 (3)	C24—C25—H25B	109.5
C11—C12—C13	123.3 (3)	H25A—C25—H25B	109.5
N2—C12—C13	108.3 (2)	C24—C25—H25C	109.5
N1—C13—C14	135.4 (2)	H25A—C25—H25C	109.5
N1—C13—C12	105.6 (2)	H25B—C25—H25C	109.5
C14—C13—C12	119.0 (2)	C24—C26—H26A	109.5
C13—C14—C9	117.1 (2)	C24—C26—H26B	109.5
C13—C14—C1	124.2 (2)	H26A—C26—H26B	109.5
C9—C14—C1	118.7 (2)	C24—C26—H26C	109.5
O3—C15—N2	127.4 (3)	H26A—C26—H26C	109.5
O3—C15—N1	126.2 (3)	H26B—C26—H26C	109.5
N2—C15—N1	106.4 (2)	C24—C27—H27A	109.5
N1—C16—C17	112.6 (2)	C24—C27—H27B	109.5
N1—C16—H16A	109.1	H27A—C27—H27B	109.5
C17—C16—H16A	109.1	C24—C27—H27C	109.5
N1—C16—H16B	109.1	H27A—C27—H27C	109.5
C17—C16—H16B	109.1	H27B—C27—H27C	109.5
H16A—C16—H16B	107.8	C24—C25'—H25D	109.5
O5—C17—O4	126.3 (3)	C24—C25'—H25E	109.5
O5—C17—C16	124.8 (3)	H25D—C25'—H25E	109.5
O4—C17—C16	108.9 (2)	C24—C25'—H25F	109.5
O4—C18—C19	110.2 (3)	H25D—C25'—H25F	109.5
O4—C18—C20	102.6 (3)	H25E—C25'—H25F	109.5
C19—C18—C20	112.3 (3)	C24—C26'—H26D	109.5
O4—C18—C21	108.3 (3)	C24—C26'—H26E	109.5
C19—C18—C21	113.2 (3)	H26D—C26'—H26E	109.5
C20—C18—C21	109.6 (3)	C24—C26'—H26F	109.5
C18—C19—H19A	109.5	H26D—C26'—H26F	109.5
C18—C19—H19B	109.5	H26E—C26'—H26F	109.5
H19A—C19—H19B	109.5	C24—C27'—H27D	109.5
C18—C19—H19C	109.5	C24—C27'—H27E	109.5
H19A—C19—H19C	109.5	H27D—C27'—H27E	109.5
H19B—C19—H19C	109.5	C24—C27'—H27F	109.5
C18—C20—H20A	109.5	H27D—C27'—H27F	109.5
C18—C20—H20B	109.5	H27E—C27'—H27F	109.5
			
O1—C1—C2—C3	12.3 (4)	C12—C13—C14—C9	−0.4 (4)
C14—C1—C2—C3	−170.7 (3)	N1—C13—C14—C1	−3.7 (5)
O1—C1—C2—C7	−165.2 (3)	C12—C13—C14—C1	177.7 (3)
C14—C1—C2—C7	11.8 (4)	C10—C9—C14—C13	0.6 (4)
C7—C2—C3—C4	−1.4 (5)	C8—C9—C14—C13	−180.0 (2)
C1—C2—C3—C4	−178.9 (3)	C10—C9—C14—C1	−177.5 (3)
C2—C3—C4—C5	1.8 (5)	C8—C9—C14—C1	1.9 (4)
C3—C4—C5—C6	−0.4 (5)	O1—C1—C14—C13	−11.4 (5)
C4—C5—C6—C7	−1.4 (5)	C2—C1—C14—C13	171.7 (3)
C3—C2—C7—C6	−0.5 (4)	O1—C1—C14—C9	166.6 (3)
C1—C2—C7—C6	177.0 (3)	C2—C1—C14—C9	−10.3 (4)
C3—C2—C7—C8	178.0 (3)	C12—N2—C15—O3	−176.3 (3)
C1—C2—C7—C8	−4.5 (4)	C22—N2—C15—O3	−8.1 (5)
C5—C6—C7—C2	1.9 (4)	C12—N2—C15—N1	3.4 (3)
C5—C6—C7—C8	−176.6 (3)	C22—N2—C15—N1	171.6 (2)
C2—C7—C8—O2	177.6 (3)	C13—N1—C15—O3	177.0 (3)
C6—C7—C8—O2	−3.9 (4)	C16—N1—C15—O3	−0.5 (4)
C2—C7—C8—C9	−4.0 (4)	C13—N1—C15—N2	−2.8 (3)
C6—C7—C8—C9	174.5 (3)	C16—N1—C15—N2	179.8 (2)
O2—C8—C9—C10	3.1 (4)	C13—N1—C16—C17	−62.9 (4)
C7—C8—C9—C10	−175.2 (3)	C15—N1—C16—C17	113.8 (3)
O2—C8—C9—C14	−176.3 (3)	C18—O4—C17—O5	9.9 (5)
C7—C8—C9—C14	5.3 (4)	C18—O4—C17—C16	−170.1 (3)
C14—C9—C10—C11	−0.9 (4)	N1—C16—C17—O5	−5.5 (4)
C8—C9—C10—C11	179.7 (3)	N1—C16—C17—O4	174.5 (2)
C9—C10—C11—C12	0.9 (4)	C17—O4—C18—C19	−65.4 (4)
C10—C11—C12—N2	179.7 (3)	C17—O4—C18—C20	174.8 (3)
C10—C11—C12—C13	−0.6 (4)	C17—O4—C18—C21	59.0 (4)
C15—N2—C12—C11	176.8 (3)	C15—N2—C22—C23	−92.2 (3)
C22—N2—C12—C11	9.2 (5)	C12—N2—C22—C23	73.9 (4)
C15—N2—C12—C13	−2.9 (3)	C24—O6—C23—O7	−6.9 (6)
C22—N2—C12—C13	−170.5 (3)	C24—O6—C23—C22	173.9 (4)
C15—N1—C13—C14	−177.8 (3)	N2—C22—C23—O7	8.9 (5)
C16—N1—C13—C14	−0.9 (5)	N2—C22—C23—O6	−171.9 (3)
C15—N1—C13—C12	1.0 (3)	C23—O6—C24—C27	41.5 (8)
C16—N1—C13—C12	177.9 (3)	C23—O6—C24—C25'	−41.3 (8)
C11—C12—C13—N1	−178.6 (3)	C23—O6—C24—C26'	−169.1 (6)
N2—C12—C13—N1	1.1 (3)	C23—O6—C24—C26	166.2 (6)
C11—C12—C13—C14	0.4 (4)	C23—O6—C24—C27'	75.5 (6)
N2—C12—C13—C14	−179.9 (2)	C23—O6—C24—C25	−79.8 (6)
N1—C13—C14—C9	178.3 (3)		

### Hydrogen-bond geometry (Å, °)

**Table:**

*D*—H···*A*	*D*—H	H···*A*	*D*···*A*	*D*—H···*A*
C16—H16B···O2^i^	0.97	2.54	3.320 (4)	137
C22—H22A···O5^i^	0.97	2.47	3.391 (3)	159
C22—H22B···O7^i^	0.97	2.29	3.190 (4)	154

Symmetry codes: (i) *x*, −*y*+3/2, *z*−1/2.
